# TACE sequential MWA guided by cone-beam computed tomography in the treatment of small hepatocellular carcinoma under the hepatic dome

**DOI:** 10.1186/s12885-023-11066-z

**Published:** 2023-06-29

**Authors:** Zhaonan Li, Kaihao Xu, Xueliang Zhou, Dechao Jiao, Xinwei Han

**Affiliations:** 1 Medical School, Southeast University, Nanjing, 210009 China; 2 Department of Interventional Radiology, The First Affiliated Hospital of Zhengzhou University, No. 1 Jianshe East Road, Zhengzhou City, 450000 Henan Province China

**Keywords:** Microwave ablation, Transcatheter arterial chemoembolization, Hepatocellular Carcinoma, Tumor ablation, Interventional radiology

## Abstract

**Purpose:**

An assessment is being conducted to determine the safety and effectiveness of using Cone-beam computed tomography (CBCT)-guided transcatheter arterial chemoembolization (TACE) and microwave ablation (MWA) sequentially to treat small hepatocellular carcinomas (HCCs) located in the hepatic dome.

**Materials and methods:**

Fifty-three patients with small HCCs in the hepatic dome who underwent TACE combined with simultaneous CBCT-guided MWA were studied. Inclusion criteria were a single HCCs ≤ 5.0 cm or a maximum of three. The safety and interventional-related complications were monitored, and local tumor progression (LTP), overall survival (OS), and prognostic factors for LTP/OS were evaluated.

**Results:**

The procedures were successfully accomplished in all patients. According to Common Terminology Criteria for Adverse Events (CTCAE), adverse reactions and complications are mainly Grade 1 or 2 (mild symptoms, no or local/noninvasive intervention indicated). Liver and kidney function and alpha-fetoprotein (AFP) levels remained within a reasonable range after 4 weeks of treatment (both p < 0.001). The mean LTP was 44.406 months (95% CI: 39.429, 49.383) and the mean OS rate was 55.157 months (95% CI: 52.559, 57.754). The combination treatment achieved 1-, 3-, and 5-year LTP rates of 92.5%, 69.6%, and 34.5%, respectively; and 1-, 3-, and 5-year OS rates of 100.0%, 88.4%, and 70.2%, respectively. Results from both univariate and multivariate Cox regression analyses showed that the tumor diameter (< 3 cm) and the distance to the hepatic dome (≥ 5 mm, < 10 mm) had a significant impact on the patient’s LTP and OS, and were related to better survival.

**Conclusion:**

CBCT-guided TACE combined with simultaneous MWA was a safe and successful treatment of HCCs located under the hepatic dome.

## Introduction

Hepatocellular Carcinoma (HCC) is a leading cause of cancer-related deaths worldwide, with a higher prevalence among elderly patients as life expectancy increases [1, 2]. The management of older HCC patients with other comorbidities will increasingly become a global issue. Data from the aging Chinese population showed that over a quarter of HCC patients and HCC-related deaths were over 70 years old [3]. Although surgical resection is still the first-line treatment for patients with small HCC, elderly patients are often deemed a high-risk group for this due to the presence of additional underlying diseases [4, 5]. Those elderly patients who undergo surgical resection tend to experience longer hospital stays, higher complication rates, and poorer overall survival (OS) than younger patients [6, 7]. Therefore, transcatheter arterial chemoembolization (TACE) combined with microwave ablation (MWA) may be a more suitable alternative for elderly patients with small HCC.

Ultrasound (US) and computed tomography (CT) have traditionally been the main image-guided modes used to perform percutaneous ablation in the treatment of HCC [8, 9]. However, due to the interference of the gas at the bottom of the lung and acoustic shadowing from the ribs, the HCC in the hepatic dome has been a blind spot for ultrasound-guided ablation [10]. Additionally, unenhanced US and CT often yield poor visualization of smaller HCC with cirrhosis in the hepatic dome. Cone-beam computed tomography (CBCT) offers multi-plane functionality, rapid image reconstruction, and superior tissue resolution, making it possible to integrate TACE and ablation processes in a single interventional procedure. After TACE, tumors can be more easily identified by CBCT due to the deposition of iodized oil. CBCT thus allows for TACE and MWA to be completed in one interventional procedure, greatly improving efficiency and reducing the duration of the interval [11, 12]. In this study, we completed TACE sequential MWA treatments on 53 small HCC patients abutting the hepatic dome.

## Materials and methods

### Patients

Patients with HCC included in this study were consistent with the diagnostic criteria of the American Association for the Study of Liver Diseases or European Association for the Study of the Liver criteria [5]. Given the retrospective nature of this project, our Institutional Review Board approved the study and waived the patient’s informed consent requirement. In this retrospective study, we included 53 patients (65.6 ± 8.9 years; range 47–79 years) who received TACE sequential MWA guided by CBCT in the treatment of small HCC under the hepatic dome. The patient characteristics are shown in **(**Table 1**)**. The inclusion and exclusion criteria are listed in **(**Table 2**)**.

**Table 1:** Inclusion and exclusion criteria

Inclusion criteria	Exclusion criteria
1 Age range: 18–75 years	Age < 18 or > 75 years
2 SHCC diagnosed according to EASL standards	No pathology or image evidence
3 Child–Pugh grade A or B	Child–Pugh grade C
3 BCLC grades are A and B	BCLC grades are C
4 ECOG score ≦ 2	ECOG score>2
4 Liver lesions > 3	The liver lesions number>3
5 Single tumor diameter ≤ 5 cm	Single tumor diameter ≧ 5 cm
6 The expected survival time>3 months	The expected survival time ≤ 3 months
7 No portal vein thrombus	Portal vein thrombus
8 No extrahepatic metastases	Extrahepatic metastases
9 PLT >40 × 109/L or PT ≤ 25 s	PLT ≤ 40 × 109/L or PT>25 s

European Association for the Study of the Liver, EASL; Eastern Cooperative Oncology Group, ECOG; platelet, PLT; prothrombin time: PT; SHCC, small hepatocellular carcinoma

**Table 2:** Patient characteristics

Characteristics	Patients (*n* = 53)	Percentage (%)
Age (Mean, range)*	65.6 ± 8.9 (47–79)	
⩾65	36	67.9%
< 65	17	32.1%
Sex		
Male	23	43.4%
Female	30	56.6%
Etiology		
Hepatitis B	34	64.1%
Hepatitis C	6	11.3%
Alcohol	9	17.0%
Unknown	4	7.6%
Liver cirrhosis		
YES	21	39.6%
NO	32	60.4%
AFP (ng/mL)		
≤ 200	14	26.4%
>200	39	73.6%
Child–Pugh class		
A	35	66.0%
B	18	34.0%
Max diameter(cm)		
< 3	41	77.4%
3⩾,<5	12	22.6%
Distance to hepatic dome (mm)		
<5	15	28.3%
⩾5,<10 mm	38	71.7%
Number of lesion		
Single(1)	24	45.3%
2–3	29	54.7%

Eastern Cooperative Oncology Group, ECOG; AFP, alpha-fetoprotein; Data are numbers of patients

*Data are mean ± standard deviation

### Procedure

#### TACE treatment

**Fig. 1:**
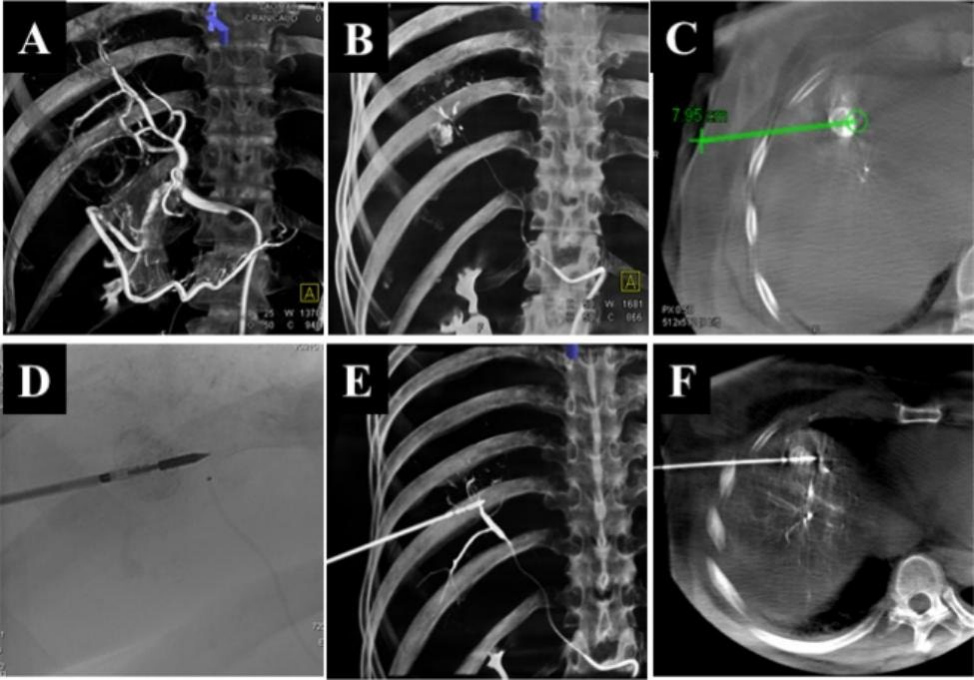
Patients with HCC with a diameter of 3 mm adjacent to the diaphragm; A. The tumor first completes the transcatheter arterial chemoembolization under the guidance of CBCT;B. HCC is marked by iodized oil; C. preoperative puncture route planning under the guidance of CBCT; D-F. The patient completes the MWA process immediately after TACE treatment

Two experienced interventional radiologists (with more than 10 years of experience each) performed all TACE procedures. First, a 5-Fr catheter was used for hepatic artery angiography to identify the tumor and its feeder(s). Subsequently, a 2.0 F microcatheter (Progreat, Terumo Corporation, Tokyo, Japan) was used for superselective catheterization of the feeding artery. Pirarubicin (THP; 60–80 mg; Shenzhen Meirui Pharmaceutical Co., Ltd. China) and iodized oil (Jiangsu Hengrui Medicine Co. Ltd., Jiangsu, China) were administered at an average dose of 10–20 mg (average 14.3 mg) and 4–6 ml (average 5.1 ml), respectively. Finally, microspheres (100–300 μm; Jiangsu Hengrui Medicine Co. Ltd., Jiangsu, China) were used for complete embolization of the artery supplying the tumor after lipiodol was evenly deposited in the tumor **(**Fig. 1**).**

### MWA treatment

All patients receiving TACE and ablation procedures were instructed to perform breathing exercises prior to treatment. Specifically, under CBCT-guided local anesthesia (Syngo-DynaCT; Siemens AG, Germany), a microwave antenna (ECO-100AI10, ECO Microwave System Co, Nanjing, China) was percutaneously inserted for tumor ablation. The patients were asked to hold their breath for 10 s, and the data collection was performed at 200° rotation and 0.37 µGy/frame X-ray dose to complete the volumetric reconstruction. iGuide VNS (Siemens puncture navigation software) was used to plan the puncture path, to align the skin entry point and the target tumor site for real-time perspective presentation, and to puncture according to the path; the power and duration of ablation were determined by the physician based on the quality of the surrounding hepatic tissue, lesion depth, and demarcation line length; usually, the tumor was ablated at 40.6 ± 0.9.7wt for 7.4 ± 2.5 min. Then, the pre- and post-ablation CT scans were superimposed to evaluate the ablation zone and any direct complications.

### Definitions and evaluation of data

The study’s primary outcome measures were overall survival (OS), Local Tumor Progression (LTP), and radiological response. OS was defined as the period between initial treatment and death from any cause. LTP was considered present if nodular enhancement was detected in the ablation area on follow-up imaging. The radiological response was evaluated using the modified response evaluation criteria in solid tumors (mRECIST; 2020 edition [13]) 4 weeks after MWA.

### Follow-up

One month post-MWA, laboratory tests such as alpha-fetoprotein (AFP) and liver function tests, as well as imaging studies including enhanced CT or enhanced MR were performed. Patients were followed up at 3-month intervals to monitor for any signs of recurrence or residuals. Using the 2020 edition of mRECIST, treatment progress was evaluated. In the event that complete response (CR) was not attained, additional treatments were carried out until CR was achieved according to the physician’s discretion.

### Statistical analysis

Statistical analysis was performed with SPSS 22.0 (SPSS Inc., Chicago, IL, USA). Categorical variables were expressed as proportions (%), and continuous variables presented as mean ± standard deviation (SD). Survival analysis was based on Kaplan–Meier curves. Univariate and multivariate Cox proportional hazards regression models were used to analyze prognostic factors for LTP and OS. A P-value of < 0.05 was considered statistically significant.

## Results

### Patient characteristics

The mean age of the patients was 65.6 ± 8.9 years (range, 47–79 years). Of the 53 patients, 17 (32.1%) were 65years old or younger, 23 (43.3%) were male, and 34 (64.1%) of the small HCC patients were associated with hepatitis B. In all of the patients, the mean target tumor size was 3.6 ± 0.9 (range, 2.6–4.9) cm, and 41 (77.4%) had tumors < 3 cm in diameter. Furthermore, 15 patients had tumors within 5 mm of the hepatic dome, and the remaining 38 patients were beyond 5 mm. Additionally, 35 (66.0%) patients were considered to be Child–Pugh A, while 18 (34.0%) were considered to be Child–Pugh B. The mean energy, ablation duration per tumor and the mean safety margin were 40.6 ± 0.9.7 kJ and 7.4 ± 2.5 min, respectively.

### Safety

**Fig. 2:**
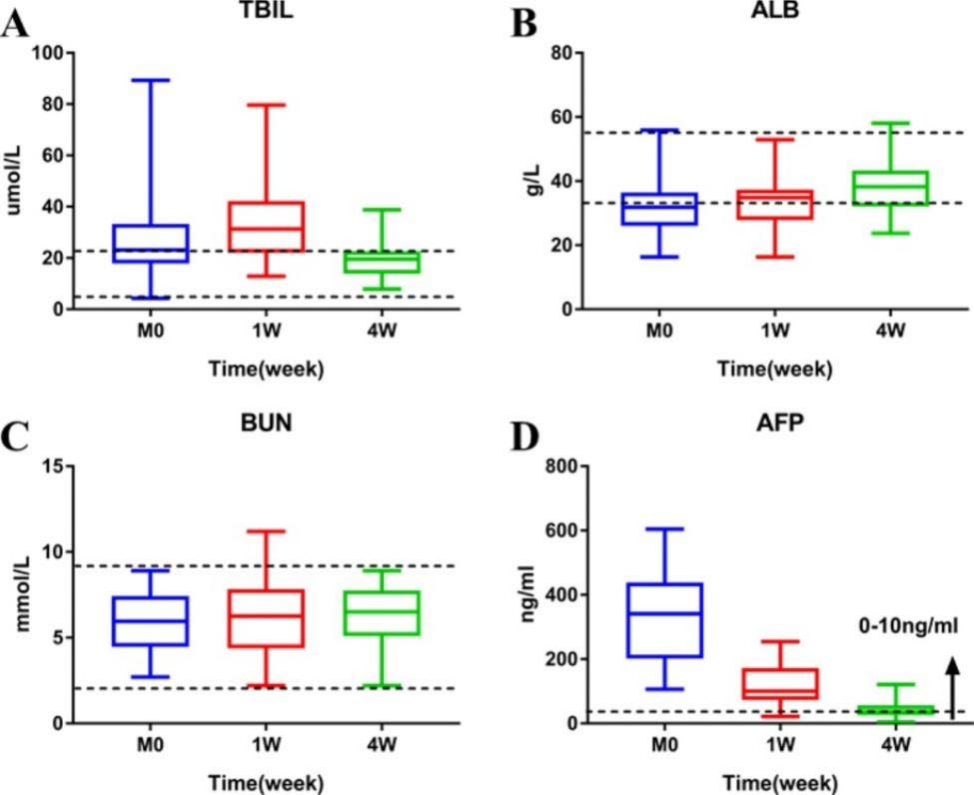
Retrospective analysis of 53 patients with TACE combined with simultaneous CBCT-guided MWA in HCC with before (M0) and after 1 week(1 W) and 4 weeks (4 W) of laboratory test results. The black dotted line indicates the normal range of laboratory inspection indicators. **(A)** Mean TBIL increased significantly at 1 W and returned to normal at 4 W; **(B)** Mean ALB increased slightly after 4 W of treatment, and most patients were in the normal range; **(C)** Mean BUN still remained normal at 1 and 4 W; **(D)** Mean AFP level decreased rapidly after treatment, and almost returned to normal level after 4 W(P<0.001); ALB, albumin; BUN, blood urea nitrogen; PT, prothrombin time; TBIL, total bilirubin; AFP ,Alpha-fetoprotein; TACE, transarterial chemoembolization; CBCT, cone-beam computed tomography; HCC, hepatocellular carcinoma; MWA, microwave ablation

All patients underwent liver/kidney laboratory tests and alpha-fetoprotein (AFP) determination over the course of the first and fourth weeks post-procedure. The mean blood urea nitrogen (BUN) stayed within its normal range for the duration of 4 weeks after treatment. However, their mean total bilirubin (TBIL; P = 0.001) level saw a slight increase within the first week after the procedure, but was brought back to its normal levels by the fourth week. The mean albumin (ALB) was also largely back in its normal range after the fourth week. Additionally, combined treatment yielded a rapid decline in AFP levels (P<0.001), and was kept within an acceptable range after 4 weeks **(**Fig. 2**)**.

### Interventional-related complications

Most adverse events and complications were CTCAE grade 1 or 2 (mild symptoms, no or local/noninvasive intervention indicated), or interventional radiology society Grade A or B (no or nominal treatment, no consequences). Exceptions included six patients (11.3%) who had localized atelectasis of the lung parenchyma caused by localized thermal injury, three patients (5.7%) with perihepatic effusion requiring thoracic drainage, and one patient (1.9%) with pneumothorax who needed closed thoracic drainage and returned to stable within 3 days after treatment. None of the patients experienced life-threatening complications during or after treatment **(**Table 3**)**.

**Table 3:** Adverse events and complications

Categories	Grades		N (%)
	CTCAE SIR		
**Adverse events**			
Fever, maximum 38 °C, no treatment	1	A	33(62.3)
Fever, > 38 °C	2	B	14(26.4)
Nausea or vomiting	2	B	16(30.2)
Mild pain, requiring nonopioid oral analgesic treatment	2	B	38(71.7)
Moderate pain, requiring opioid oral analgesic treatment	2	B	10(18.9)
Mild liver dysfunction, requiring conservative treatment	2	B	21(39.6)
Total bilirubin elevation, transient	2	B	8(15.1)
Hypoalbuminemia, transient	1	A	2(3.8)
liver abscess	2	B	1(1.9)
**complications**			
transient lung injury	3	B	6(11.3)
Pleural effusion	3	B	3(5.7)
pneumothorax	4	D	1(1.9)

National Cancer Institute Common Terminology Criteria for Adverse Event (CTCAE version 4.03),

Society of Interventional Radiology (SIR) classification system for Complications. Data are numbers of events. Data in parentheses are percentages

### LTP and OS

**Fig. 3:**
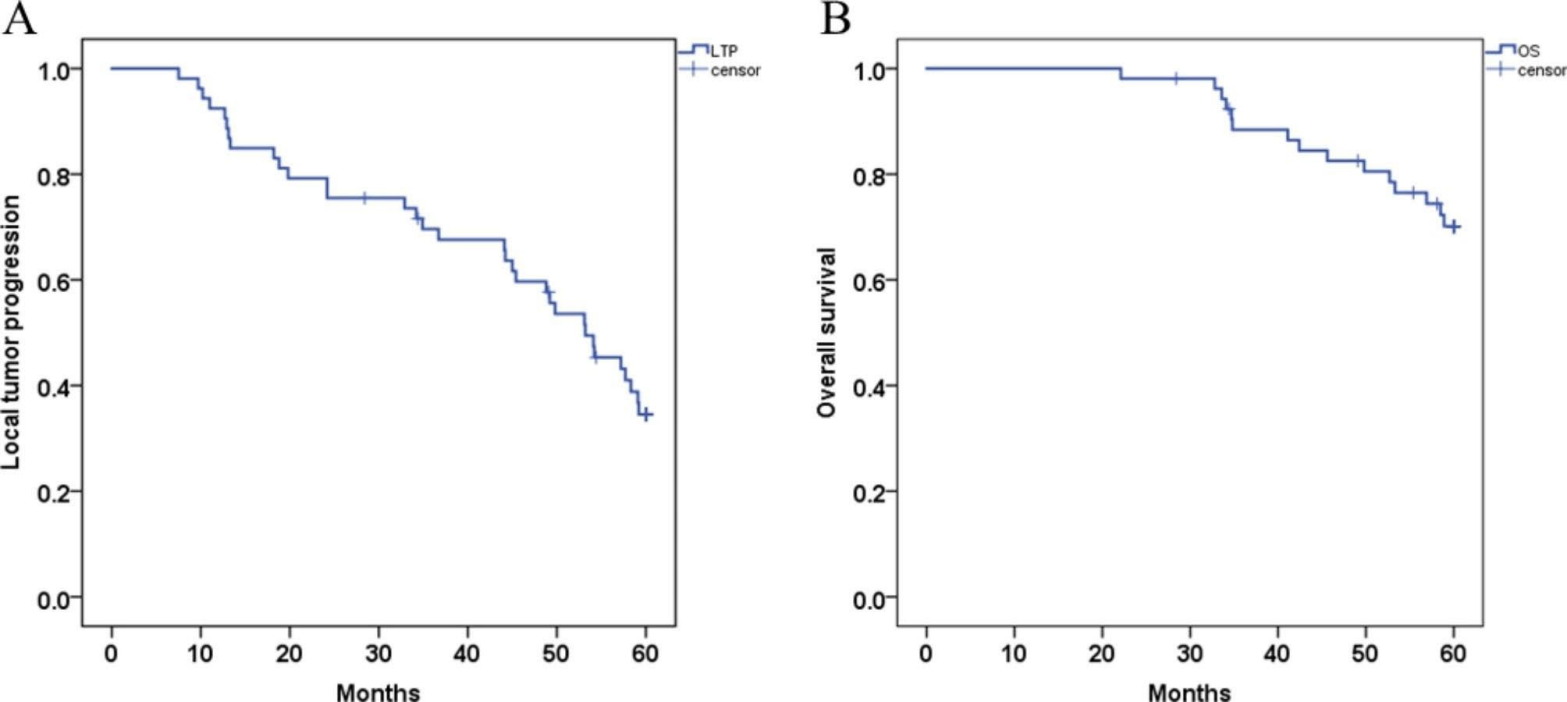
Mean Local tumor progression (LTP) was 44.406 months (95% CI: 39.429, 49.383) and mean overall survival (OS) rates was 55.157 months (95% CI: 52.559, 57.754) in the combination therapy; **A**; The 1-, 3-, and 5-year LTP rates of combination group were 92.5%, 69.6% and 34.5%, respectively; **B**. The 1-, 3- and 5-year OS rates were 100.0%, 88.4% and 70.2%, respectively; LTP, Local tumor progression; OS, overall survival;

The survival analysis of CBCT-guided TACE sequential MWA for the treatment of small HCCs under the hepatic dome revealed a mean LTP of 44.406 months (95% CI: 39.429, 49.383) and mean OS of 55.157 months (95% CI: 52.559, 57.754) in the combination therapy. The 1-, 3- and 5-year LTP rates of the combination treatment were 92.5%, 69.6% and 34.5%, respectively **(**Fig. 3A**)**; the 1-, 3- and 5-year OS rates were 100.0%, 88.4% and 70.2%, respectively **(**Fig. 3B**)**. Univariate Cox proportional hazard regression indicated that Child-Pugh (A vs. B), liver cirrhosis (YES vs. NO) and the number of lesions (single vs. 2–3 lesions) were not associated with longer LTP and OS (both P > 0.05). Additionally, both univariate and multivariate Cox regression revealed that the tumor diameter (< 3 cm) and the distance to hepatic dome (⩾10 mm, <5 mm) did have a significant impact on the patient’s LTP and OS and were related to better survival **(**Table 4**)**.

**Table 4:** Factors affecting LTP and OS

Parameters	LTP			*P*	OS			*P*
	HR	95%CI			HR	95%CI		
		Lower Higher				Lower Higher		
**Univariate Cox regression**								
Age(≥ 65 vs<65)	1.410	0.680	2.923	0.355	2.198	0.796	6.070	0.129
Child-Pugh (A vs. B)	1.390	0.690	2.801	0.356	1.671	0.606	4.610	0.321
Liver cirrhosis (YES vs. NO)	1.344	0.670	2.694	0.405	1.022	0.364	2.872	0.967
Number of lesion (single vs. 2–3 lesions)	1.803	0.882	3.688	0.106	1.473	0.524	4.142	0.463
Max diameter(< 3 cm VS 3 cm⩾,<5 cm)	5.317	2.452	11.532	**0.000**	6.503	2.324	18.192	**0.000**
Distance to hepatic dome(< 5mmVS ⩾5 mm,<10 mm)	27.074	9.152	80.094	**0.000**	19.482	5.367	70.726	**0.000**
**Multivariate Cox regression**								
Age (≥ 65 vs<65)	1.725	0.369	2.324	0.871	0.783	0.130	4.705	0.789
Child-Pugh (A vs. B)	1.725	0.801	3.716	0.164	2.038	0.579	7.182	0.268
Liver cirrhosis (YES vs. NO)	2.855	1.251	6.511	0.013	1.451	0.336	6.267	0.618
Number of lesion (single vs. 2–3 lesions)	2.943	1.302	6.652	0.009	2.106	0.643	6.897	0.218
Max diameter(< 3 cm VS 3 cm⩾,<5 cm)	4.322	1.502	12.434	**0.007**	3.421	0.918	12.747	**0.067**
Distance to hepatic dome(< 5mmVS ⩾5 mm,<10 mm)	31.338	9.338	105.178	**0.000**	17.189	4.271	69.173	**0.000**

**OS** overall survival, **HR** hazard ratio, **CI** confidence interval, **LTP** local tumor progression

### Tumor diameter and the distance to hepatic dome

**Fig. 4:**
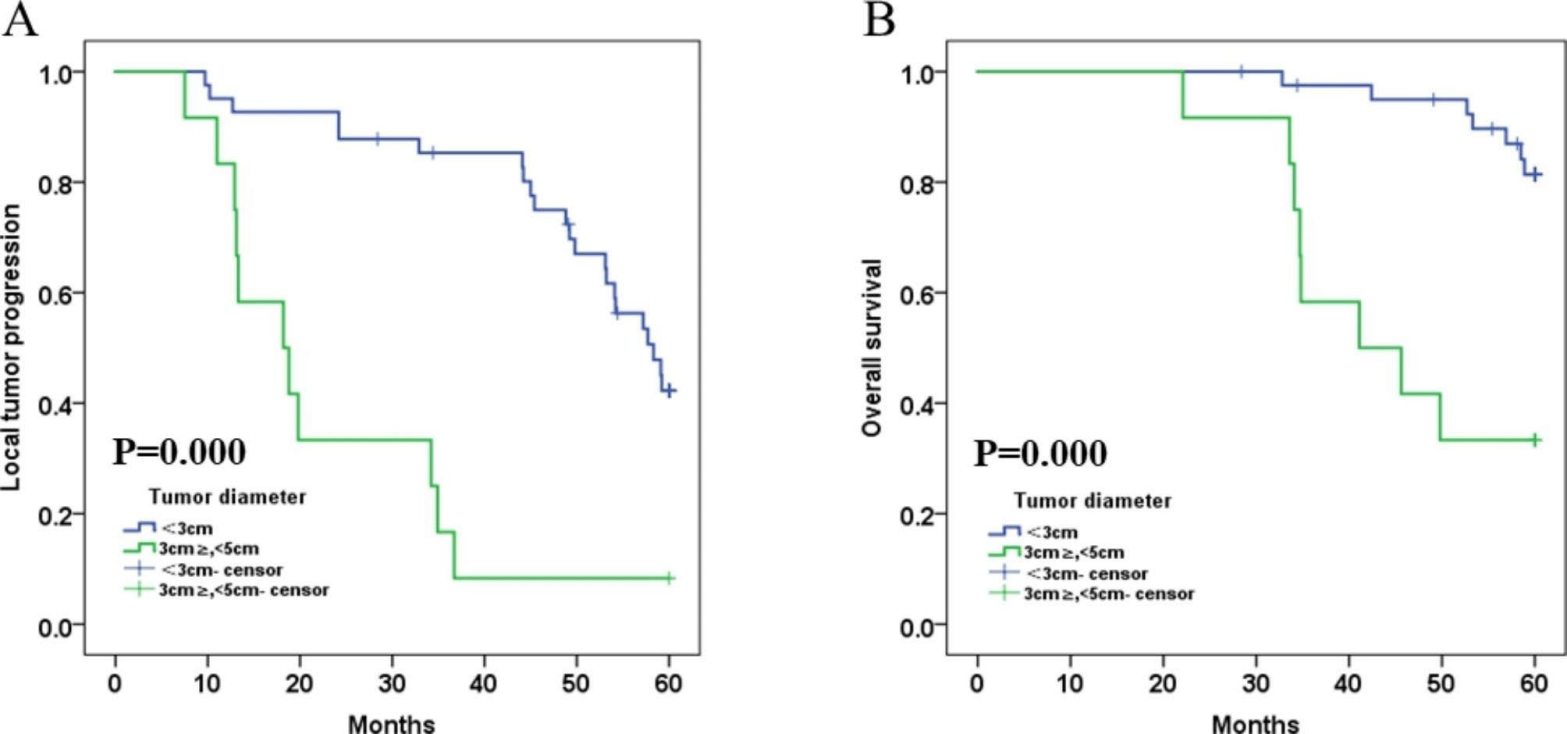
** A.** Comparison of LTP between tumor diameter<3 cm and tumor diameter ≥ 3 cm,<5 cm after TACE sequential MWA treatment. The mean LTP was 50.622 months (95% CI: 46.183, 55.060) for procedures with tumor diameter<3 cm versus 23.367 months (95% CI: 15.116, 31.617) for procedures with tumor diameter ≥ 3 cm,<5 cm (*p* = 0.000, log-rank test). The 1-, 3-, and 5-year LTP-free survival rates in patients with tumor diameter<3 cm were 95.1%, 85.3% and 42.2%, respectively, and the 1-, 3- and 5-year LTP-free survival rates in patients with tumor diameter ≥ 3 cm,<5 cm were 83.3%, 8.3% and 8.3%, respectively; **B.** Comparison of OS between tumor diameter<3 cm and tumor diameter ≥ 3 cm,<5 cm after TACE sequential MWA treatment. The mean OS was 58.342 months (95% CI: 56.732, 59.952) for procedures with tumor diameter<3 cm versus 44.650 months (95% CI: 37.492, 51.808) for procedures with tumor diameter ≥ 3 cm,<5 cm (*p* = 0.000, log-rank test). The 1-, 3-, and 5-year OS rates in patients with tumor diameter<3 cm were 100.0%, 97.5% and 81.3%, respectively, and the 1-, 3- and 5-year OS rates in patients with tumor diameter ≥ 3 cm,<5 cm were 100.0%, 58.3% and 33.3%, respectively

**Fig. 5:**
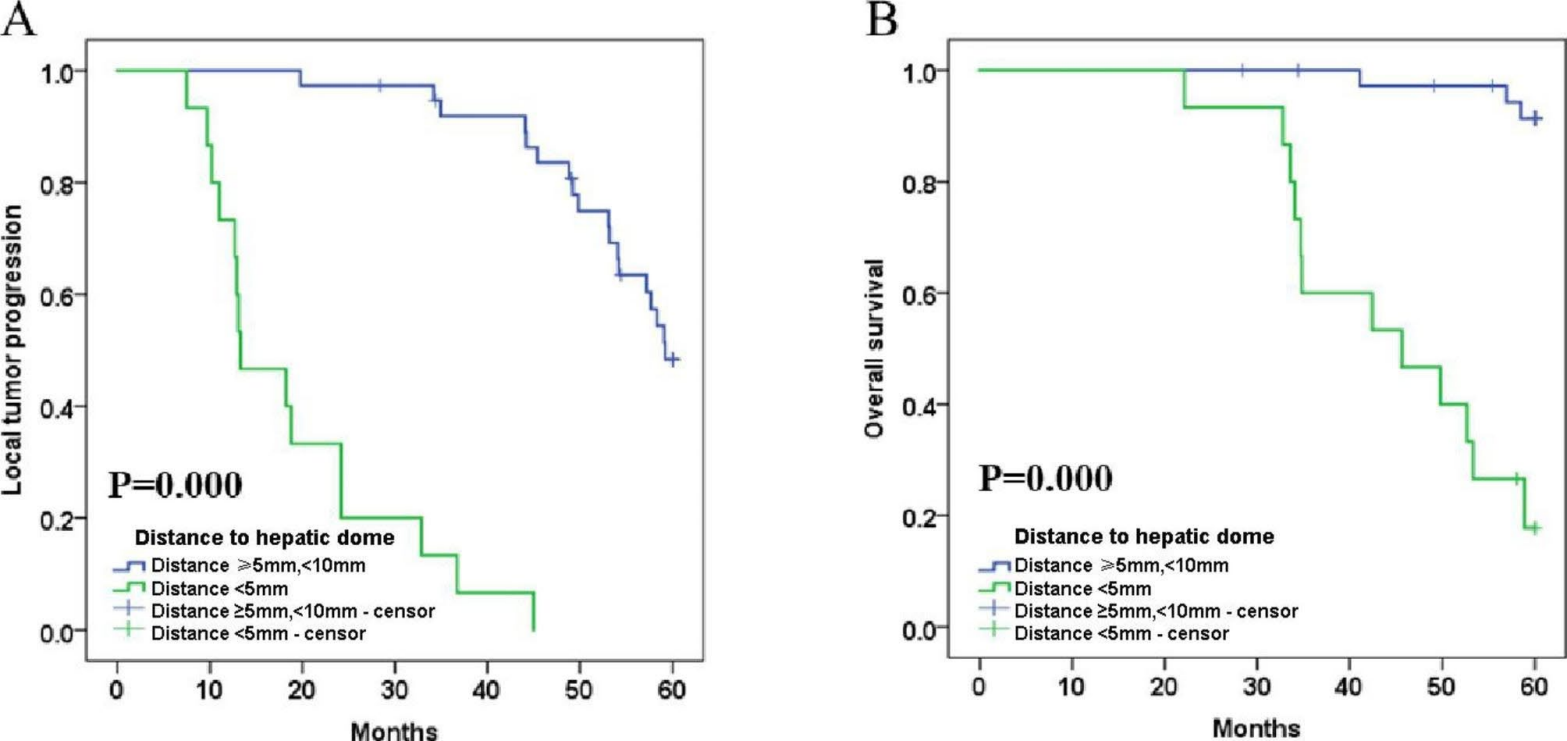
** A.** Comparison of LTP between HCC distance to hepatic dome<5 mm and distance ≥ 5 mm,<10 mm after TACE sequential MWA. The mean LTP was 19.360 months (95% CI: 13.719, 57.263) for procedures with HCC distance to hepatic dome <5 mm versus 54.350 months (95% CI: 51.437, 57.263) for procedures with distance ≥ 5 mm,<10 mm (*p* = 0.000, log-rank test). The 1-, 3-, and 5-year LTP-free survival rates in patients with HCC distance to hepatic dome<5 mm were 73.3%, 6.7% and 0.0%, respectively, and the 1-, 3- and 5-year LTP-free survival rates in patients with distance ≥ 5 mm,<10 mm were 100.0%,91.9% and 48.3%, respectively; **B.** Comparison of OS between HCC distance to hepatic dome<5 mm and distance ≥ 5 mm,<10 mm after TACE sequential MWA. The mean OS was 44.962 months (95% CI: 38.906, 51.019) for procedures with HCC distance to hepatic dome <5 mm versus 59.339 months (95% CI: 58.314, 60.365) for procedures with distance ≥ 5 mm,<10 mm (*p* = 0.000, log-rank test). The 1-, 3-, and 5-year OS rates in patients with HCC distance to hepatic dome<5 mm were 100.0%, 60.0% and 17.8%, respectively, and the 1-, 3- and 5-year OS rates in patients with distance ≥ 5 mm,<10 mm were 100.0%, 100.0% and 91.3%, respectively

The mean LTP for procedures with tumor diameter < 3 cm was 50.622 months (95% CI: 46.183, 55.060), compared to 23.367 months (95% CI: 15.116, 31.617) for those with tumor diameter ≥ 3 cm, <5 cm (p = 0.000, log-rank test). The 1-, 3- and 5-year LTP-free survival rates for patients with tumor diameter < 3 cm were 95.1%, 85.3% and 42.2%, respectively, and those with tumor diameter ≥ 3 cm, <5 cm were 83.3%, 8.3% and 8.3%, respectively **(**Fig. 4A**)**. The mean OS was 58.342 months (95% CI: 56.732, 59.952) for those with tumor diameter < 3 cm, and 44.650 months (95% CI: 37.492, 51.808) for those with tumor diameter ≥ 3 cm, <5 cm (p = 0.000, log-rank test). The 1-, 3- and 5-year OS rates for patients with tumor diameter < 3 cm were 100.0%, 97.5% and 81.3%, respectively, and those for tumor diameter ≥ 3 cm, <5 cm were 100.0%, 58.3% and 33.3%, respectively **(**Fig. 4B**)**. Regarding procedures with HCC distance to hepatic dome < 5 mm, the mean LTP was 19.360 months (95% CI: 13.719, 57.263); for those with distance ≥ 10 mm, <5 mm, it was 54.350 months (95% CI: 51.437, 57.263) (p = 0.000, log-rank test). The 1-, 3- and 5-year LTP-free survival rates for patients with HCC distance to hepatic dome < 1 mm were 73.3%, 6.7% and 0.0%, respectively; for those with distance ≥ 10 mm, <5 mm they were 100.0%, 91.9% and 48.3%, respectively **(**Fig. 5A**)**. The mean OS for procedures with HCC distance to hepatic dome < 5 mm was 44.962 months (95% CI: 38.906, 51.019), compared to 59.339 months (95% CI: 58.314, 60.365) for those with distance ≥ 10 mm, <5 mm (p = 0.000, log-rank test). The 1-, 3- and 5-year OS rates for patients with HCC distance to hepatic dome < 5 mm were 100.0%, 60.0% and 17.8%, respectively, and those for distance ≥ 10 mm, <5 mm were 100.0%, 100.0% and 91.3%, respectively **(**Fig. 5B**)**.

## Discussion

With the increased life expectancy in many countries, the conventional management model for HCC is not appropriate for elderly patients [14]. While surgical resection is considered a reasonable first-line treatment for small HCC, the long-term benefits of radical resection for elderly patients remain unclear due to the impacts of compromised liver function or regenerative capacity, portal hypertension, tumor location, and comorbidities [15–18]. For small HCC, TACE combine thermal ablation are usually not recommended as a single ablation is equally effective [19]. However, for tumor located close to the diaphragm, it is difficult to determine the puncture path under conventional ultrasound and CT equipment [20]. The 3-year LTP for small HCC near the diaphragm treated with ablation alone is as high as 62%, whereas TACE combined with thermal ablation for small HCC near the diaphragm has shown promising therapeutic effects, with a 5-year LTP rate of only 3% [21–24]. Therefore, most centers adopt a combination of TACE and thermal ablation as the generally accepted resection alternative for small HCC patients near the diaphragm.

Radiofrequency ablation (RFA) and MWA are commonly employed thermal ablation techniques for hepatic malignancies. In comparison to RFA, MWA has similar benefits such as larger volume of necrosis, shorter procedure time, and quicker attainment of higher temperatures, and is less affected by heat-sink effects from adjacent vasculature [25, 26]. A propensity score analysis of MWA and RFA for the treatment of perivascular HCC demonstrated similar disease control rates in both groups (94% vs. 91%, p = 0.492). Moreover, MWA had better control of tumor progression for periportal HCC or single-nodule perivascular HCC patients compared to RFA [27]. In a meta-analysis of MWA and RFA for HCC showed no difference in terms of complete response (risk ratio (RR) 1.01, 95% CI 0.99–1.02). The local recurrence rate was similar between MWA and RFA, but MWA had significantly lower distant recurrence rate (RR 0.60, 0.39–0.92) [28]. Moreover, the study of TACE combined with either RFA or MWA for the treatment of HCC indicated that TACE + MWA (TM) group had better overall survival (hazard ratio [HR]: 1.55; 95% confidence interval [CI]: 1.09–2.21, p = 0.01) and higher rate of complete response (RR: 0.87; 95% CI: 0.79–0.96, p = 0.003) than TACE + RFA (TR) group. The advantage of TM was greater for those with tumor diameter less than 3 cm [29].

Despite its widespread acceptance in clinical centers, conventional computed tomography (cCT)-guided MWA is limited in its ability to accurately delineate the precise location of tumors and the boundaries of ablation lesions [31]. Multiple contrast agent administrations can further increase the burden on the kidneys. As an alternative, CBCT-guided TACE sequential MWA is a reliable treatment option [32]. The first TACE procedure facilitates the deposition of iodine-containing oil inside the tumor and the utilization of CBCT-mounted flat detector technology for improved spatial resolution to acquire richer CT information and real-time fluorescence imaging for the guidewire to realign the puncture angle, direction and depth in accordance with the precise location of the lesion. This approach allows for the completion of two treatments in a single procedure without transferring the patient to the CT room, thereby reducing the interval between the two treatments and minimizing the patient’s risk. In this retrospective research, satisfactory results were obtained from CBCT-guided TACE sequential MWA treatment of small HCC in the hepatic dome. The mean LTP was 44.406 months (95% CI: 39.429, 49.383) and the mean OS was 55.157 months (95% CI: 52.559, 57.754). The LTP rate was 92.5%, 69.6%, and 34.5% at 1, 3, and 5 years, respectively, while the OS rate was 100.0%, 88.4%, and 70.2% at 1, 3, and 5 years, respectively.

Although CBCT imaging can provide high-quality spatial resolution, poor density resolution is a major problem, which can make it difficult to accurately visualize the extent of tumor ablation during treatment. To address these issues, we primarily utilize the following methods to evaluate the degree of tumor ablation: (1) Using CBCT perfusion imaging after MWA, complete ablation was indicated when there was no abnormal staining around the lesion. (2) Adjusting the window width range to 120–350 HU and the window level range to 25–45 HU for visual observation after ablation. (3) Immediately pre- and post-ablation CT scans were superimposed to evaluate the ablation zone and any direct complications.

However, our study presents several limitations that should be taken into consideration. Firstly, the study is retrospective and the small sample size increases the possibility of bias. Secondly, CBCT imaging has its own challenges, such as the density of iodinated oil causing artifacts, as well as breath holding and immobility being necessary for successful image reconstruction. Moreover, only tumors with well-defined borders were selected as target lesions, disregarding those surrounded by streak artifacts from catheters or located in truncated segments of the liver, which may affect the accuracy of the results. Nevertheless, we have ensured the effectiveness of the study conclusions by strict inclusion and exclusion criteria, rational statistical methods, and high-quality follow-up data. Thus, to provide better treatment decisions for small hepatocellular carcinoma located in the hepatic dome.

## Conclusion

In summary, CBCT-guided TACE sequential MWA treatment of small HCCs under the hepatic dome has demonstrated to be of clinical value. Furthermore, CBCT can be used to guide accurate puncture, which would assist in the decision-making process for interventional procedures and improve the safety of the treatment by minimizing associated risks.

## Data Availability

The datasets used and analysed during the current study are available from the corresponding author on reasonable request.
